# Prediction of Water Chemical Oxygen Demand with Multi-Scale One-Dimensional Convolutional Neural Network Fusion and Ultraviolet–Visible Spectroscopy

**DOI:** 10.3390/biomimetics10030191

**Published:** 2025-03-20

**Authors:** Jingwei Li, Yijing Lu, Yipei Ding, Chenxuan Zhou, Jia Liu, Zhiyu Shao, Yibei Nian

**Affiliations:** School of Electrical, Energy and Power Engineering, Yangzhou University, No. 88 South University Road, Yangzhou 225009, China; mz120231217@stu.yzu.edu.cn (Y.L.); mz120221197@stu.yzu.edu.cn (Y.D.); 005151@yzu.edu.cn (Y.N.)

**Keywords:** UV-Vis spectroscopy, COD, one-dimensional convolutional neural network

## Abstract

Chemical oxygen demand (COD) is a critical parameter employed to assess the level of organic pollution in water. Accurate COD detection is essential for effective environmental monitoring and water quality assessment. Ultraviolet–visible (UV-Vis) spectroscopy has become a widely applied method for COD detection due to its convenience and the absence of the need for chemical reagents. This non-destructive and reagent-free approach offers a rapid and reliable means of analyzing water. Recently, deep learning has emerged as a powerful tool for automating the process of spectral feature extraction and improving COD prediction accuracy. In this paper, we propose a novel multi-scale one-dimensional convolutional neural network (MS-1D-CNN) fusion model designed specifically for spectral feature extraction and COD prediction. The architecture of the proposed model involves inputting raw UV-Vis spectra into three parallel sub-1D-CNNs, which independently process the data. The outputs from the final convolution and pooling layers of each sub-CNN are then fused into a single layer, capturing a rich set of spectral features. This fused output is subsequently passed through a Flatten layer followed by fully connected layers to predict the COD value. Experimental results demonstrate the effectiveness of the proposed method, as it was compared with three traditional methods and three deep learning methods on the same dataset. The MS-1D-CNN model showed a significant improvement in the accuracy of COD prediction, highlighting its potential for more reliable and efficient water quality monitoring.

## 1. Introduction

Water pollution is a significant environmental issue that has wide-ranging impacts on life, health, agricultural safety, and environmental protection [1]. The rapid pace of global industrialization and the extensive use of organic fertilizers have led to the discharge of wastewater containing both industrial and organic pollutants into surface waters [2]. Organic pollution is one of the primary contributors to surface water contamination, posing a serious threat to aquatic ecosystems. In particular, the presence of severe organic pollution can lead to a sharp decline in dissolved oxygen levels in water. This depletion threatens aquatic life and disrupts the natural balance of the water environment. Prolonged exposure to organic pollutants has been linked to various health risks, including an increased likelihood of cancer and developmental deformities. Therefore, it is essential to implement reliable monitoring systems to track and assess organic pollution in water [3]. Chemical oxygen demand (COD) serves as a crucial indicator for measuring organic pollution. COD quantifies the amount of oxygen consumed during the oxidation and decomposition of organic compounds under controlled conditions [4,5]. The ability to detect COD rapidly and accurately is critical for effective daily water quality monitoring, sewage treatment, and the tracking of pollution sources.

COD in water can be determined through a variety of methods, including chemical, biological, and physical approaches [6]. Spectroscopy is a commonly employed physical method for identifying substances in water [7,8]. It works by creating quantitative spectral models that can be employed to analyze the water’s composition. Traditional chemical methods for COD analysis, while effective, often require long processing times and the application of various reagents, which can complicate the procedure. In contrast, spectroscopic methods offer several advantages, especially when combined with advanced data processing and modeling techniques. These methods provide fast responses, high detection efficiency, and superior precision compared to traditional techniques. Furthermore, spectroscopic methods allow for in situ and real-time measurement, making them ideal for continuous water quality monitoring [9]. Among the various spectroscopic techniques, UV-Vis spectroscopy has emerged as the most widely applied method for evaluating water quality due to its effectiveness and ease of application in detecting a wide range of pollutants.

Establishing an accurate relationship between spectral data and COD values is crucial after obtaining spectral data. This relationship forms the foundation for reliable COD detection and quantification. Recent research has increasingly focused on leveraging advanced machine learning techniques to model this relationship, addressing errors in traditional COD detection methods [10,11]. For example, Wang et al. [12] applied turbidity compensation on UV-Vis spectra and employed a partial least squares (PLS) method to develop a COD prediction model. Their model demonstrated superior performance compared to existing methods. Similarly, Rong et al. [13] developed a two-step ozone dosing control model employing Linear Discriminant Analysis (LDA) and Support Vector Machine (SVM), achieving a high prediction accuracy of 94.29% for ozonation needs, thus optimizing the ozonation process. Lepot et al. [14] compared five methods: linear regression, SVM, evolutionary algorithm, PLS, and others on datasets from sewers, rivers, and wastewater treatment plants (WWTPs) for TSS, COD, and dissolved oxygen. The results showed that PLS and SVM were the most effective methods, based on the Kruskal–Wallis test and RMSE values. In another study, Cao et al. [15] explored UV absorbance for rapid COD determination in aquaculture wastewater. They compared four calibration methods: multiple linear regression (MLR), PLS, LS-SVM, and BP-ANN. Additionally, they introduced a variable selection method, the successive projections algorithm (SPA), to select effective wavelengths. Six different spectral pretreatment methods were compared. The best prediction result was obtained by the BP-ANN model based on the 11 EWs with the optimum spectral pretreatment method of the standard normal variate, and the coefficient of determination of prediction was 0.90, the root mean squared error of prediction was 10.96 mg/L, and the residual predictive deviation was 5.06. Despite these advancements, traditional machine learning algorithms still face challenges when dealing with the complex, nonlinear relationships between spectra and COD. These traditional methods often require significant feature preprocessing and can suffer from poor detection accuracy and generalization ability. In contrast, deep learning has emerged as a promising alternative due to its capacity to autonomously extract features and learn directly from the data without the need for extensive feature engineering. The layered structure of deep learning methods allows them to better handle various complexity problems [16,17,18].

Deep learning has become increasingly prominent in various fields, largely replacing traditional methods due to its ability to automatically extract features and perform exceptionally well across a wide range of tasks. The recent success of deep learning techniques can be attributed to their remarkable performance improvements over conventional approaches [19,20]. Deep learning has proven to be particularly advantageous in the context of COD detection. For instance, Guan et al. [21] enhanced traditional detection models by incorporating deep learning and a spectrum preprocessing algorithm. They introduced a novel COD detection network to address the issue of poor model generalization. This model demonstrated excellent performance, exhibiting both noise tolerance and high accuracy, thereby improving the reliability of COD predictions. Similarly, Mia et al. [22] proposed a new method for spectrometric COD quantification, by a double-channel one-dimensional convolutional neural network (CNN), to process UV-Vis absorption spectra data. Their CNN model outperformed the traditional PLS model, reducing the relative residual mean square error percentage (RRMSEP) from 4.5% to 1.3% for COD solutions. Additionally, regression accuracy increased from 68% to 91%, underscoring the model’s effectiveness. Convolutional neural networks (CNNs) possess several advantages over traditional models, including superior self-learning and self-adaptive capabilities. CNNs are particularly adept at handling nonlinear problems [23,24]. It makes them well suited for complex prediction tasks like COD detection. Furthermore, CNNs require minimal spectral preprocessing compared to traditional methods, as their inherent feature extraction and local feature mapping abilities significantly reduce the need for manual data preprocessing, leading to more efficient and accurate predictions [25,26].

This paper focuses on improving the accuracy of COD detection in water by combining CNNs with UV-Vis spectroscopy. UV-Vis spectra, typically one-dimensional sequences, present a unique challenge in spectral analysis. Directly applying a two-dimensional CNN to these one-dimensional data would require significant time and effort to convert the data from one-dimensional to a two-dimensional format. Furthermore, such a transformation might lead to a misalignment of features, potentially resulting in redundant or irrelevant features that could degrade the model’s prediction accuracy. To address these challenges, this paper proposes an optimization strategy where the neural network structure is specifically designed to handle long one-dimensional UV-Vis absorption spectra data effectively. The proposed method involves a multi-scale one-dimensional convolutional neural network (MS-1D-CNN) fusion model, which is tailored to extract spectral information at multiple scales. The model captures diverse features from the spectra by multiple convolution kernels of different sizes, helping to reduce prediction errors and enhance accuracy. The effectiveness of the proposed MS-1D-CNN model is rigorously evaluated and compared with traditional methods, demonstrating its superior performance in COD prediction for water samples. This comparison highlights the advantages of the proposed model in improving the precision of environmental monitoring.

## 2. Materials and Methods

### 2.1. Dataset

Water samples for this paper were collected from the Ancient Canal in Yangzhou, a historically significant waterway with over 2000 years of history. However, the canal faces significant water quality challenges due to the increasing discharge of wastewater from urban development, agricultural runoff, and industrial activities. These sources of pollution have contributed to a decline in water quality, making effective monitoring and testing critical for ensuring the safety and sustainability of local water resources. Water quality monitoring plays an essential role in supporting local production activities and the daily lives of residents. Water samples were collected continuously from various regions of the canal over one year, from June 2023 to June 2024. Sampling was conducted twice daily, excluding holidays, yielding a total of 480 samples. To evaluate the performance of the proposed COD prediction model, the collected data were split into a training/calibration set and a testing/prediction set at a ratio of 5:1. To maintain a similar distribution between the two sets, an equidistant sampling method was applied. The dataset was first sorted in ascending order based on COD standard values. Every sixth sample was selected for the testing set, while the remaining samples were assigned to the training set. Consequently, the sequence numbers of the testing set samples were 3, 9, 15, …, and 477. This approach resulted in a training/calibration set of 400 samples and a testing/prediction set of 80 samples. These division results are commonly used in machine learning applications. The COD standard values for the collected samples were determined by the rapid digestion spectrophotometric method (HJ/T399-2007), a reliable and widely recognized chemical method [27]. The determination was carried out by a DRB200 digester device and a DR3900 visible spectrophotometer (Hach, Loveland, CO, USA), both of which are commonly employed in environmental testing for their precision and accuracy in measuring COD levels. The COD statistical results of the sample set are shown in Table 1.

**Figure 1:**
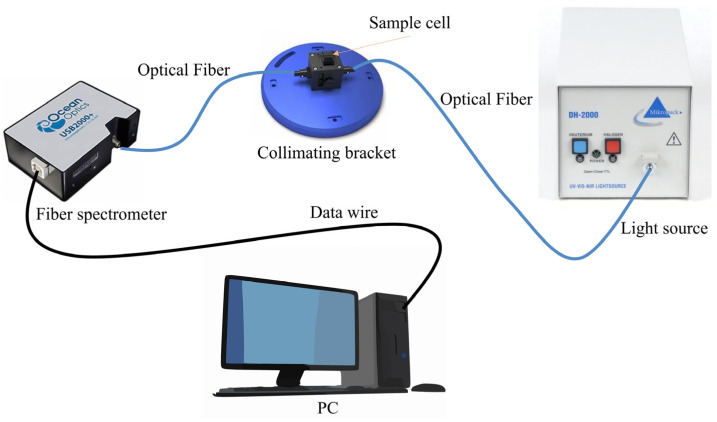
UV-Vis spectroscopy collecting system.

**Figure 2:**
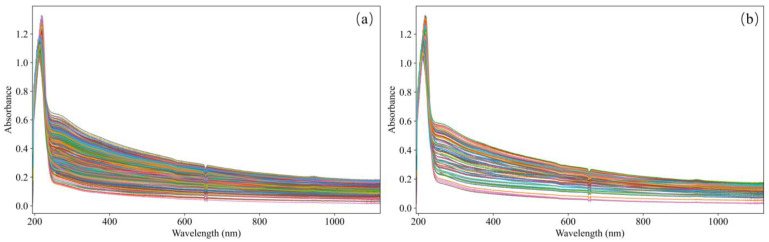
UV-Vis spectroscopy of the water samples. (**a**) UV-Vis spectroscopy of training set; (**b**) UV-Vis spectroscopy of testing set.

The spectral data in this paper are collected through a UV-Vis spectroscopy system, as shown in Figure 1. The system is composed of several key components that work together to obtain high-quality spectral data. The light source employed in the system is a DH-2000 deuterium–halogen light source, which emits light across a wide range of wavelengths, from 190 to 2500 nm, ensuring that the system can capture the full spectrum of interest for accurate COD detection (Ocean Optics, Orlando, FL, USA). The sample cell, which holds the water sample for analysis, has an optical path length of 10 mm, a standard length for achieving reliable absorbance measurements. The spectra are recorded by a USB2000+ spectrometer (Ocean Optics, Orlando, FL, USA), which is connected to the computer and operates in conjunction with OceanView software 2.0.8 to store spectra within the wavelength range of 193.91 to 1121.69 nm (Ocean Optics, Orlando, FL, USA). A computer is used to store and process the acquired spectra, as well as to construct predictive models through appropriate algorithms and software. This UV-Vis spectroscopy system was employed to collect spectral data from the water samples, providing detailed information on the absorbance characteristics of each sample. A total of 480 spectra were collected from different water samples, and these spectra are shown in Figure 2, serving as the basis for further analysis and model development in this paper.

### 2.2. The Proposed Method

#### 2.2.1. Multi-Scale One-Dimensional Convolutional Neural Network

**Figure 3:**
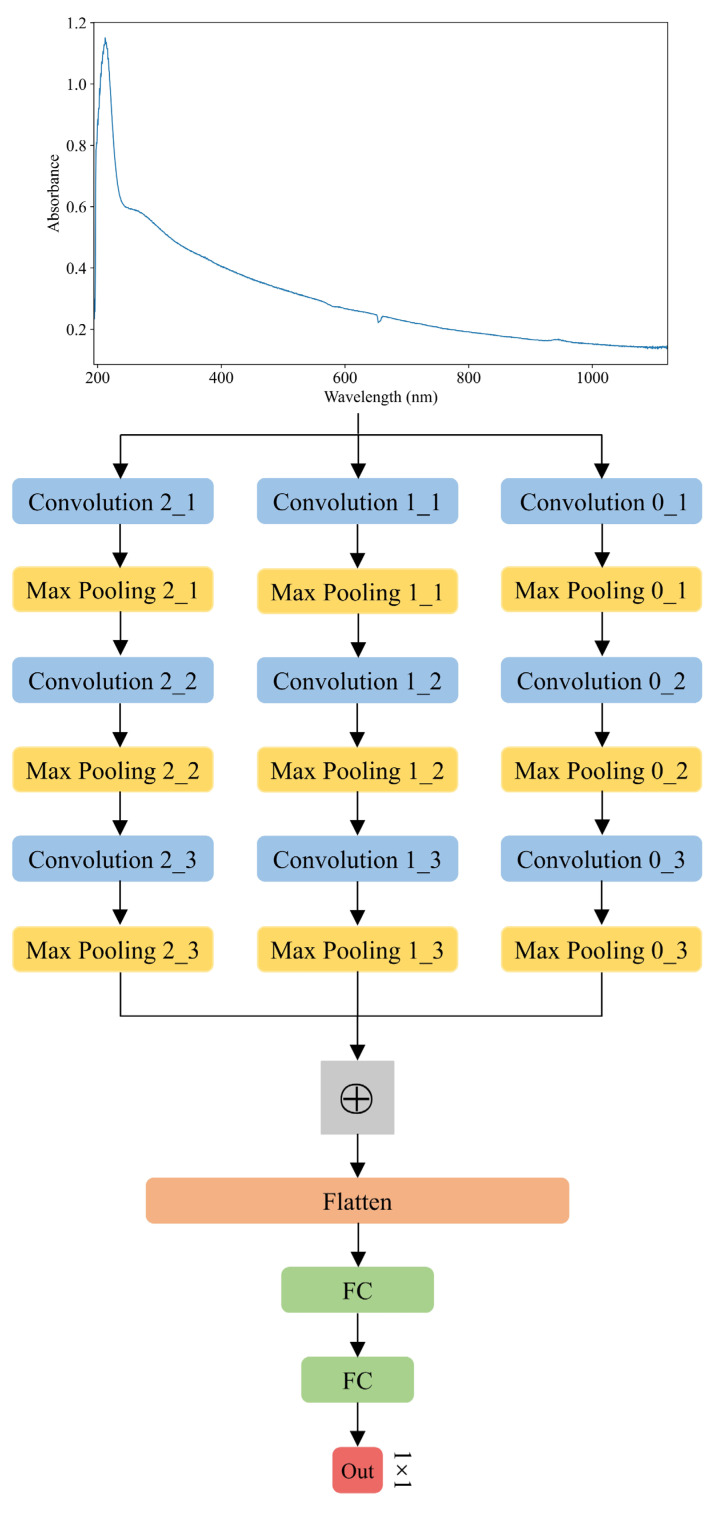
Multi-scale one-dimensional convolutional neural network.

The proposed model for UV-Vis spectroscopy modeling and COD prediction is a multi-scale one-dimensional convolutional neural network (MS-1D-CNN). This model is designed to capture and learn spectral features from the UV-Vis spectra more effectively than traditional methods. The MS-1D-CNN consists of three parallel sub-networks, each of which is a one-dimensional convolutional neural network (1D-CNN) with a different convolution kernel size. These varying kernel sizes enable the network to learn spectral features at different scales, enhancing the model’s ability to capture detailed and diverse information in the spectral data. The extracted features from each 1D-CNN are then fused in a feature fusion layer, which integrates the information from different scales. The fused features are subsequently passed through fully connected (FC) layers to make the final COD prediction. One of the key advantages of employing a multi-scale CNN is its ability to capture more spectral information compared to traditional CNNs, which typically focus on a single scale. The MS-1D-CNN architecture includes several key components: convolutional layers that perform the initial feature extraction, pooling layers that reduce dimensionality, feature fusion layers that combine information from different scales, a Flatten layer that prepares the data for the fully connected layers, and fully connected layers that perform the final prediction. The main structure of the MS-1D-CNN is shown in Figure 3. The specific parameters of the model will be determined through experiments in Section 3.1. This multi-scale approach enhances the model’s ability to make accurate COD predictions based on UV-Vis spectra.

(1) Convolutional Layer.

A traditional neural network is typically composed of three layers: an input layer, a hidden layer, and an output layer. Each of these layers contains a significant number of neurons, and the neurons between adjacent layers are fully connected, meaning that each neuron in one layer is connected to every neuron in the subsequent layer. However, one issue with traditional neural networks is the large number of parameters that they have. This can result in gradient dispersion during training, which makes the network harder to optimize and can negatively affect the learning process. Convolutional layers help address this issue by reducing the number of parameters in the network, which not only improves the learning efficiency but also helps prevent overfitting [28]. The convolutional layer, in particular, plays a critical role in CNNs by allowing the network to automatically learn hierarchical features from the input data. In traditional image processing tasks, two-dimensional convolution is commonly employed to extract features from image data. However, in this paper, the input data consist of one-dimensional spectral vector data, for which one-dimensional convolution is more appropriate. This type of convolution helps to capture the essential features of the spectral data while maintaining computational efficiency and ensuring that relevant information is learned from the one-dimensional input.

**Figure 4:**
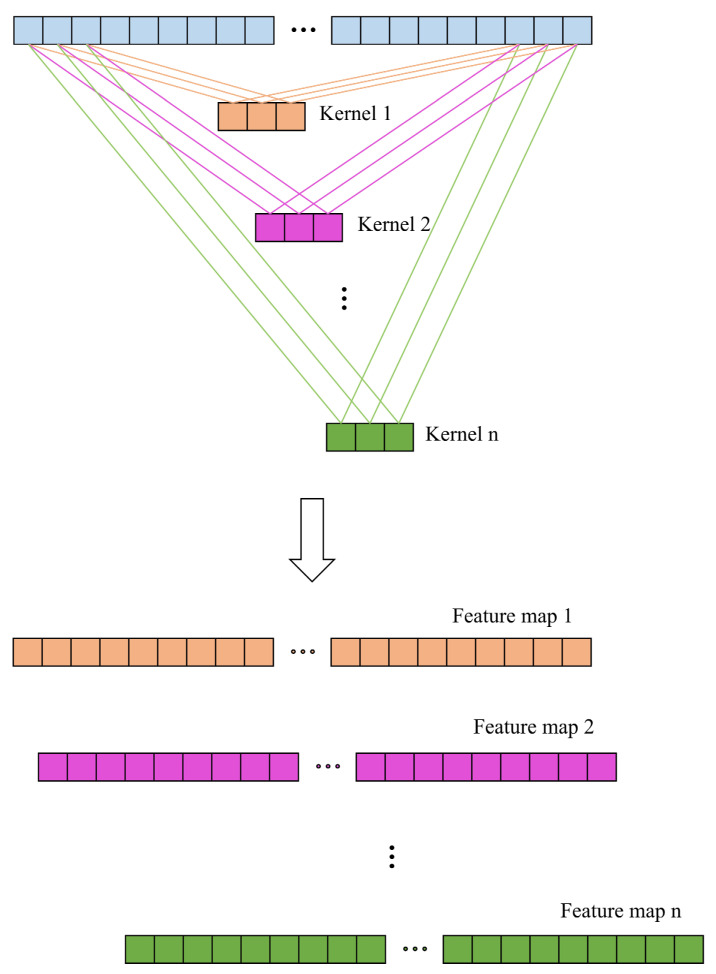
One-dimensional convolution operation diagram.

The input spectral data are first fed into the convolutional layer, where the convolutional operation begins. During this operation, the convolutional kernel processes only a small part of the input data at a time, moving across the entire input in a sliding window manner. Initially, the kernel starts at the left side of the input data, and the parameters of the kernel are multiplied by the corresponding values in the input data’s coverage area. These multiplied values are then summed up to produce a single output, as shown in Equation (1). Once the calculation is complete for the current region, the kernel moves to the right to perform the convolution on the next part of the input data. The parameters within the convolutional kernel are initially randomly generated, and during the learning process, these parameters are adjusted to minimize the error. In the proposed model, one-dimensional convolutions are applied with multiple kernel sizes, enabling the network to capture spectral features at different scales, such as short, medium, and long distances. To introduce nonlinearity into the network and improve its learning capacity, the output of the convolution is passed through an activation function. The ReLU activation function, which is widely used in CNNs, is applied after the convolution [29]. A diagram that visually illustrates the convolution operation can be found in Figure 4, providing a clear understanding of the process.

(1) $$y^{j}=f\left(\sum_{i=1}^{n}\omega^{ij}*x^{i}+b^{i}\right)$$

where * is the convolution operation; $y^{j}$ is the layer j output feature map; $x^{i}$ is the layer i input feature map; $\omega^{ij}$ is the convolution kernel in this layer; and $b^{i}$ is the bias of the layer i feature map.

(2) Pooling layer

The pooling layer is positioned immediately after the convolutional layer, where it takes the output from the convolution layer as its input. The primary purpose of the pooling layer is to reduce the amount of information output from the convolutional layer by performing down-sampling. This operation helps to reduce the number of parameters or the dimensionality of the feature map, thereby improving the overall efficiency of the model during training. Pooling ensures that the learned features are more representative and accurate by retaining only the most essential features within local regions. While similar to the convolutional layer in that it also employs a kernel, the pooling kernel differs in that it does not contain any parameters. The pooling operation begins at the left side of the input data, and the pooling kernel moves across the data in a manner similar to the convolutional kernel. Max pooling is the most commonly used technique in CNNs, as it selects the most prominent feature within each local region [30]. In this paper, one-dimensional max pooling is employed to extract the most important spectral features from the input data. The pooling kernel has a size of 1 × 2, with a stride set to 2, ensuring that the pooling operation effectively reduces the dimensionality while preserving key information.

(3) Feature fusion layer

The proposed method adopts a multi-scale feature extraction strategy by employing three parallel sub-1D-CNNs, each with a different kernel size. This approach allows the network to capture feature maps at varying scales, providing a more comprehensive representation of the spectral data. Each sub-1D-CNN is responsible for extracting and learning features at its respective scale. Once the features are extracted by the individual sub-networks, their outputs are combined into a single layer through feature fusion. The fusion process is performed by element-wise addition, which effectively merges the information from all three scales into a unified feature map, as shown in Equation (2). To further enhance the quality of the learned features, normalization is applied to each dimension of the fused feature map. This step adjusts the increasingly skewed data distribution, aiming to standardize the data and bring them into a more uniform distribution. Normalization plays a critical role in addressing gradient vanishing issues, which can hinder the learning process. Additionally, normalization helps to improve both the training speed and the generalization ability of the model. The normalization procedure is expressed as Equation (3).

(2) $$F=M_{0}^{3}+M_{1}^{3}+M_{2}^{3}$$

(3) $$f=\frac{F-\mu }{\sigma }$$

where ⨁ is the element-wise addition; $M_{0}^{3}$, $M_{1}^{3}$, and $M_{2}^{3}$ are multi-scale features; F is the fused feature; μ is the mean value; σ is the standard deviation; f is the fusion feature after standardized.

(4) Flatten layer

The Flatten layer serves a crucial role in neural network architectures by converting a multi-dimensional input into a one-dimensional output [31]. This transformation is essential for the subsequent processing of the data in fully connected layers. The Flatten layer is commonly applied as a bridge between the convolutional layers and the fully connected layers in CNNs. After the feature maps have been processed and reduced by the convolutional and pooling layers, the data typically remain in a multi-dimensional form. The Flatten layer reshapes these data into a single long vector, allowing them to be fed into the fully connected layers, which are designed to perform high-level reasoning and prediction. Thus, the Flatten layer plays a fundamental role in preparing the data for further processing within the network, ensuring the smooth flow of information across different layers of the model.

(5) Fully connected layer

The fully connected layer plays a vital role in preparing the final output of a CNN. After the convolutional and pooling layers have extracted and learned features from the input data, the fully connected layer maps the learned feature distribution to the label space, facilitating the transition from feature learning to prediction [32]. Specifically, while the convolutional layers map the input data to the feature space of the hidden layers, the fully connected layer takes these learned features and maps them to the output space. In a fully connected layer, every neuron is connected to all neurons in the preceding layer, allowing for the comprehensive integration of all the learned features. This dense interconnection enables the network to learn complex information and relationships within the data. The final output produced by the fully connected layer is then passed to the end layer, which generates the model’s predictions. In the case of COD prediction, the output of the last fully connected layer is a single value, which corresponds to the predicted COD value for a given input spectrum.

#### 2.2.2. Model Training

The loss function plays a crucial role in the training and optimization of CNNs by quantifying the error between the predicted output and the actual target values. This error serves as the foundation for adjusting the model’s parameters, guiding the network toward improved predictions. During the training process, the error is backpropagated from the final layer to the preceding layers through the backward propagation algorithm. This allows the model to adjust the parameters of the network, which are essential for feature learning and prediction. After each backpropagation step, the parameters are updated, and these adjustments are employed in subsequent training iterations. This iterative process continues in a loop until the loss function value is minimized or the maximum number of epochs is reached, ensuring the model converges to an optimal solution. In this paper, the model employs the mean square error (MSE) as the loss function, which measures the average squared difference between the predicted and actual COD values. MSE is commonly used for regression tasks as it provides a straightforward and effective way to quantify prediction accuracy, enabling the network to learn and adapt efficiently during the training stage. The MSE is shown in Equation (4).

(4) $$MSE=\frac{1}{n}\sum_{i=1}^{n}{\left(y_{i}-{\hat{y}}_{i}\right)}^{2}$$

where $y_{i}$ is the measured value according to the standard method; ${\hat{y}}_{i}$ is the prediction value of the model; n is the number of samples.

The optimization of the proposed model is achieved through the Adam optimization algorithm, a popular choice due to its adaptive learning rate and efficiency in training deep neural networks [33]. The model is trained for a maximum of 200 epochs, ensuring sufficient iteration for the network to learn and fine-tune its parameters while avoiding overfitting. The experimental setup is conducted with PyTorch GPU 2.5.1, taking advantage of dual Geforce RTX 4090 GPUs (Taiwan Semiconductor Manufacturing Company, Taiwan, China) to accelerate the training process. The environment also includes CUDA 11.8 for parallel processing, a 2.9 GHz Intel^®^ Xeon^®^ Silver 4310 CPU (Intel’s semiconductor fabrication plants, Chandler, AZ, USA), and 32 GB of RAM for general computations. The operating system employed is Windows 10, with Python 3.9.6 as the programming language, ensuring compatibility and stability in running the model training and evaluation processes.

### 2.3. COD Prediction Process of the Proposed Method

**Figure 5:**
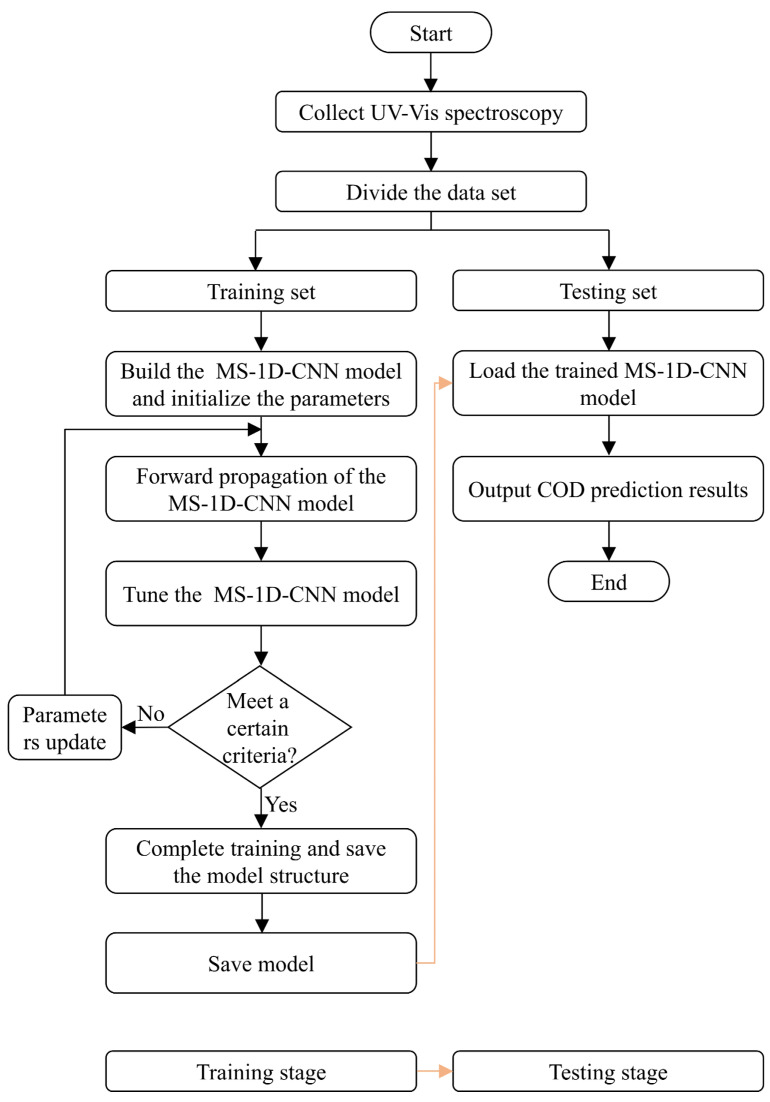
COD prediction process of the proposed method.

The process of COD prediction is built upon the MS-1D-CNN model, which leverages UV-Vis spectroscopy data for accurate forecasting. The model is designed to automatically and adaptively extract features from the UV-Vis spectra, ensuring that relevant spectral information is effectively captured for COD prediction. A key strength of the proposed MS-1D-CNN model is its superior generalization capability, which allows it to perform well on spectral datasets. The entire process of the proposed method is systematically shown in Figure 5.

(1) The UV-Vis spectroscopy of the water samples is collected by a dedicated spectral collecting system, designed to capture the absorption spectra across a wide range of wavelengths, as shown in Figure 1. The collected spectra serve as the essential input for the MS-1D-CNN model, which is designed to process and analyze the spectral data for COD prediction.

(2) The input samples are randomly divided into two distinct subsets: a training set and a testing set, ensuring a fair evaluation of the model’s performance. The division follows a 5:1 ratio. The training set is employed during the model training stage, where the model learns to recognize relationships within the data. This testing set is crucial for assessing the model’s generalization capability and its performance on data that were not included in the training process.

(3) The MS-1D-CNN model is trained. The training process of the MS-1D-CNN model involves two key operations: forward propagation and backward propagation. In forward propagation, the input data are passed through the network, where they are processed layer by layer. Each layer applies specific operations to extract relevant features and generate intermediate outputs. The error between the predicted and standard values is then employed in backward propagation to update the model’s parameters. This process is repeated iteratively, with the model adjusting its parameters to minimize the error and improve its performance. Once the model has been trained to meet a predefined criterion, the model is saved.

(4) Once the MS-1D-CNN model has been trained and saved, it is ready for application in the testing stage. The first step in this stage is loading the saved model, which contains the optimized parameters learned during training. After loading the model, the testing set is input into the network. This ensures that the model’s performance can be evaluated on unseen data, providing a reliable assessment of its generalization capability.

### 2.4. Performance Indices

The performance of various models for COD prediction can vary significantly, with each model offering distinct advantages and disadvantages depending on the context of the application. To make meaningful comparisons between these models, it is essential to use standardized evaluation indices that provide a consistent and objective basis for assessment. In this paper, the model performance is evaluated by three key performance indices: the coefficient of determination (R^2^), the root mean squared error of calibration (RMSEC), and the root mean squared error of prediction (RMSEP). These indices are critical for determining how well the model fits the data and its predictive accuracy. Specifically, a higher R^2^ value and lower RMSEC/RMSEP values indicate superior model performance, while a lower R^2^ value and higher RMSEC/RMSEP values indicate poorer performance. The equations for calculating these performance indices are provided in Equations (5)–(7).

(5) $$R^{2}=1-\frac{\sum_{i=1}^{n}{\left({\hat{y}}_{i}-y_{i}\right)}^{2}}{\sum_{i=1}^{n}{\left(y_{i}-\bar{y}\right)}^{2}}$$

(6) $$RMSEC=\sqrt{\frac{1}{n^{c}-1}\sum_{i=1}^{n^{c}}{\left({\hat{y}}_{i}^{c}-y_{i}^{c}\right)}^{2}}$$

(7) $$RMSEP=\sqrt{\frac{1}{n^{p}}\sum_{i=1}^{n^{p}}{\left({\hat{y}}_{i}^{p}-y_{i}^{p}\right)}^{2}}$$

where $y_{i}$ is the measured value based on the standard method; $\bar{y}$ is the average of $y_{i}$; ${\hat{y}}_{i}$ is the prediction value of the model; n is the number of samples in the calibration/prediction set; $y_{i}^{c}$ is the measured value of the calibration set based on the standard method; ${\hat{y}}_{i}^{c}$ is the prediction value of the calibration set based on the model; $n^{c}$ is the number of samples in the calibration set; $y_{i}^{p}$ is the measured value of the prediction set based on the standard method; ${\hat{y}}_{i}^{p}$ is the prediction value of the prediction set based on the model; and $n^{p}$ is the number of samples in the prediction set.

## 3. Experiments and Results Analysis

### 3.1. Selection of Model Parameters

The proposed MS-1D-CNN model consists of three parallel sub-networks, each designed as a 1D-CNN with a distinct convolution kernel size. This multi-scale structure enhances feature extraction by capturing patterns at different scales. To improve the model’s generalization and robustness, an ensemble learning approach is employed. The ensemble learning framework optimizes the parameters of MS-1D-CNN by leveraging the optimal settings derived from individual 1D-CNN models. For initialization, the model parameters follow the PyTorch default initialization tool to ensure stable training. The convolution operation is configured with a step size of 1, while padding is adjusted based on the kernel size to maintain the original feature shape. Pooling operations are performed with a kernel size of 2 and a step size of 2 to reduce feature dimensionality while preserving essential information. The fully connected layers consist of two layers with 128 and 32 neurons, respectively, allowing for effective feature transformation before output. Adam is selected as the optimizer, with a learning rate of 0.0001 to ensure smooth convergence. The training process is conducted with a batch size of 20 and spans 200 epochs to facilitate comprehensive learning. Several key parameters significantly influence the model’s performance. These include the convolutional kernel size, the number of convolutional kernels, and the number of convolutional layers. Additionally, the number of neurons in the fully connected layers and the depth of the fully connected layers also impact predictive accuracy. In this study, these parameters will be systematically optimized through experimental analysis to achieve the best performance.

(1) Convolutional kernel size

The convolutional kernel size plays a crucial role in feature extraction within a 1D-CNN. Small kernels focus on capturing fine-grained local features, which are essential for detecting subtle variations in spectral data. In contrast, large kernels encompass a broader range of regional information, allowing the model to capture long-range dependencies. Selecting an appropriate kernel size is critical for optimizing model performance. In this study, the dataset consists of UV-Vis spectroscopy data with a fixed length of 2048. To comprehensively analyze the impact of kernel size, multiple convolutional kernel sizes were tested. The sizes examined include 1, 3, 5, 7, 9, 11, 21, 51, and 101. Throughout these experiments, key structural parameters were kept constant to ensure a fair comparison. Specifically, the number of convolutional layers was set to 3, and each layer contained 32 convolutional kernels. The primary objective of this analysis was to investigate how different convolutional kernel sizes influence the prediction performance of COD in the 1D-CNN. The experimental results provide insights into the relationship between kernel size and predictive accuracy. A detailed summary of the findings is presented in Table 2.

As shown in Table 2, The convolution kernel size has a significant impact on the performance of COD prediction in the 1D-CNN model. An inappropriate kernel size may lead to suboptimal performance. Therefore, selecting the most effective kernel size is essential for optimizing the model. To evaluate the influence of different kernel sizes, the RMSEC was used as a performance metric. The ranking of the kernel sizes, based on RMSEC values, followed this order: 5, 3, 1, 7, 9, 101, 11, 51, and 21. Smaller kernel sizes (5, 3, and 1) yielded the lowest RMSEC, indicating superior predictive performance. Based on these findings, the MS-1D-CNN model assigns the top three kernel sizes, 5, 3, and 1, to its three parallel 1D-CNN sub-networks. This selection ensures that the model captures both fine local patterns and broader spectral features.

(2) Number of convolutional kernels

The number of convolution kernels plays a crucial role in feature extraction and representation in a 1D-CNN model. Each convolution kernel acts as a filter, detecting specific patterns in the input data. A higher number of convolution kernels increases the number of output channels in the feature map, allowing the model to capture a more diverse set of spectral features. This enhancement improves the model’s ability to distinguish subtle variations in COD-related spectral patterns. However, there is a trade-off when selecting the number of convolution kernels. While increasing the number of kernels enhances feature richness, it also raises the total number of parameters and computational complexity. Excessive kernels may lead to overfitting, where the model becomes too specialized to the training data and loses generalization capability. Therefore, finding the optimal balance is essential for improving predictive performance while maintaining computational efficiency. After determining the optimal convolution kernel size, this study further investigated the influence of the number of convolution kernels on COD prediction accuracy. To ensure a controlled evaluation, the convolution kernel size was fixed at 3 for all experiments in this analysis. The experimental findings, including the influence of different kernel quantities on model performance, are summarized in Table 3.

As shown in Table 3, the number of convolution kernels significantly influences the performance of COD prediction in the 1D-CNN model. An optimal selection of convolution kernels ensures a balance between feature richness and computational efficiency. To evaluate the influence of different kernel quantities, the RMSEC was used as a performance metric. The ranking of convolution kernel numbers, based on RMSEC values, follows this order: 32, 16, 64, 8, 128, 512, and 256. The results indicate that 32 convolution kernels achieved the lowest RMSEC, demonstrating the best predictive performance. The final selection of convolution kernel numbers is determined based on both RMSEC performance and the number of convolutional layers in the MS-1D-CNN model. This ensures that the model maintains an optimal balance between accuracy and computational efficiency.

(3) Number of convolutional layers

The number of convolutional layers in a 1D-CNN plays a pivotal role in the model’s ability to extract hierarchical features. Increasing the number of layers enhances the network’s capacity to capture more complex patterns from the input data, thereby improving its feature extraction capabilities. However, adding more layers also increases the number of parameters in the model, which in turn raises computational complexity. Excessively deep networks may become prone to overfitting, as they risk memorizing the training data rather than generalizing to unseen samples. Therefore, a careful balance between depth and model complexity is crucial for optimal performance. After determining the optimal convolution kernel size and the number of convolution kernels, this study further investigated the influence of the number of convolution layers on COD prediction accuracy. To ensure a controlled evaluation, the convolution kernel size was fixed at 3 and the number of convolution kernels was fixed at 32 for all experiments in this analysis. The experimental results, which highlight the influence of convolutional layers on model performance, are presented in Table 4.

As shown in Table 4, the number of convolutional layers significantly influences the performance of COD prediction in the 1D-CNN model. It is essential to consider the trade-off between increased depth and computational complexity, as too many layers can lead to overfitting and hinder generalization. To evaluate the influence of different convolutional layer quantities, the RMSEC was used as a performance metric. The ranking of the convolution layer quantities, based on RMSEC values, followed this order: 3, 2, 9, 4, 10, 7, 8, 5, 6, and 1. The results suggest that the model with 3 convolutional layers achieved the best performance, followed by networks with two and nine layers. Based on this ranking, the MS-1D-CNN model selected 3 convolutional layers for each of its 1D-CNN sub-networks. This choice ensured that the model struck an effective balance between feature extraction and computational efficiency.

(4) Number of neurons in the fully connected layers

The fully connected layer in a 1D-CNN plays a crucial role in integrating the features learned by the convolutional layers and producing the final output. The number of neurons in the fully connected layers directly influences the model’s ability to capture complex relationships between the extracted features. A higher number of neurons can increase the model’s capacity to represent intricate patterns and improve performance. However, there is a trade-off associated with increasing the number of neurons. More neurons lead to an increase in the number of parameters, which can raise the risk of overfitting. Therefore, it is essential to carefully select the number of neurons in the fully connected layers to achieve optimal performance without compromising generalization. After determining the optimal convolution kernel size, the number of convolution kernels, and the number of convolutional layers, this study further investigated the influence of the number of neurons in the fully connected layers on COD prediction accuracy. To ensure a controlled evaluation, the convolution kernel size was fixed at 3, the number of convolution kernels was fixed at 32, and the number of convolutional layers was fixed at 3 for all experiments in this analysis. The experimental results, detailing the relationship between the number of neurons in the fully connected layers and model performance, are presented in Table 5.

As shown in Table 5, the number of neurons in the fully connected layers plays a pivotal role in determining the performance of COD prediction in the 1D-CNN model. The optimal number of neurons must strike a balance between sufficient complexity and the risk of overfitting. To evaluate the influence of different neuron quantities, the RMSEC was used as a performance metric. The ranking of the number of neurons in the fully connected layers, based on RMSEC values, followed this order: 256, 32, 64, 512, 16, 4096, 128, and 1024. The results show that 256 neurons yield the best performance, followed by 32 and 64 neurons. The final selection of the optimal number of neurons was made based on the number of fully connected layers.

(5) Number of fully connected layers

The number of fully connected layers in a 1D-CNN significantly impacts the model’s ability to capture complex, nonlinear relationships between the extracted features. A balance must be struck between the number of fully connected layers and the risk of overfitting. After determining the optimal convolution kernel size, the number of convolution kernels, the number of convolutional layers, and the number of neurons in the fully connected layers, this study further investigated the influence of the number of fully connected layers on COD prediction accuracy. To ensure a controlled evaluation, the convolution kernel size was fixed at 3, the number of convolution kernels was fixed at 32, the number of convolutional layers was fixed at 3, and the number of neurons in the fully connected layers was fixed at 256 for all experiments in this analysis. The results, which provide insights into how the number of fully connected layers influences model performance, are presented in Table 6.

As shown in Table 6, the number of fully connected layers in a 1D-CNN model plays a crucial role in determining the performance of COD prediction. Therefore, selecting an optimal number of fully connected layers is essential for balancing predictive accuracy and computational efficiency. To determine the optimal number of fully connected layers, the RMSEC was used as a performance metric. The ranking of the number of fully connected layers, based on RMSEC values, follows this order: 2, 1, 3, 5, and 4. Based on this ranking, the optimal number of fully connected layers for the MS-1D-CNN model is set to 2. This configuration balances model complexity and performance, minimizing the risk of overfitting while maintaining high predictive accuracy.

The final parameters of the MS-1D-CNN model are determined based on extensive experimental analysis. The finalized parameters are summarized in Table 7, providing a detailed overview of the model configuration. The MS-1D-CNN consists of three parallel 1D-CNN sub-networks, each designed to capture different feature scales. The selected convolution kernel sizes for these sub-networks are 1, 3, and 5. This multi-scale approach enhances the model’s ability to extract both fine-grained and broader spectral features, improving the overall predictive accuracy. Each 1D-CNN sub-network contains 3 convolutional layers to ensure effective feature extraction. The number of convolution kernels in these layers is set to 16, 32, and 64, respectively. This configuration allows the network to progressively capture more complex patterns while balancing model complexity and computational cost. At the final stage, two fully connected layers integrate the extracted features and generate the model’s output. The first fully connected layer consists of 256 neurons, facilitating robust feature fusion. The second fully connected layer contains 32 neurons, refining the learned representations before producing the final prediction. This architecture ensures both high accuracy and computational efficiency, making the MS-1D-CNN suitable for COD prediction based on UV-Vis spectroscopy.

### 3.2. Training Procedure

Adjusting hyperparameters is a critical step in optimizing deep learning models. The proper selection of hyperparameters directly affects model performance, training stability, and convergence speed. In this study, two key hyperparameters were analyzed: learning rate and batch size. These parameters play a fundamental role in determining the effectiveness and efficiency of model training. The learning rate is an essential hyperparameter in optimization algorithms. It controls the step size for updating model parameters during backpropagation. A learning rate that is too high can cause unstable training, leading to divergence. Conversely, a learning rate that is too low may result in slow convergence or becoming stuck in local minima. Selecting an appropriate learning rate is crucial for achieving optimal performance. The batch size defines the number of samples used to compute gradients in a single training iteration. A small batch size allows for more frequent updates, potentially improving model generalization but increasing training noise. A large batch size stabilizes updates and can speed up training on powerful hardware, but it may require careful tuning to avoid convergence issues. Balancing batch size is essential for efficient model training. The influence of the learning rate and batch size on training effectiveness and speed is systematically analyzed. Different combinations of these hyperparameters are tested to evaluate their influence on model convergence and performance. The experimental results, including the training performance for various learning rate and batch size settings, are presented in Table 8.

The selection of learning rate and batch size has a significant influence on COD prediction performance in deep learning models. A well-chosen learning rate ensures stable and efficient training, while an appropriate batch size balances computational efficiency and model generalization. To systematically evaluate the influence of these hyperparameters, the RMSEC was used as the primary performance metric. The results reveal that the optimal combination is a learning rate of 0.0001 and a batch size of 32. This configuration achieves the best balance between training stability, convergence speed, and prediction accuracy.

**Figure 6:**
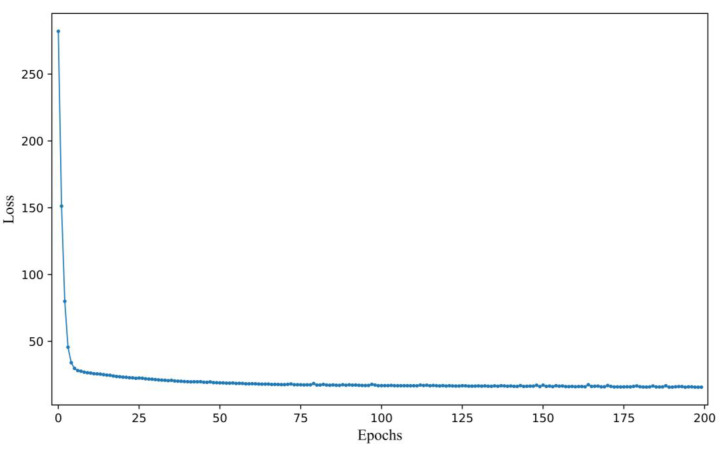
Changes in loss values during the training process of the MS-1D-CNN model.

The Adam optimization algorithm was employed to optimize the network parameters during the training process. This algorithm is widely known for its efficiency in adjusting the weights of the network in order to minimize the loss error. To ensure that the best-performing model was employed during the testing stage, the model was saved at the end of each epoch, as shown in Figure 6. The loss value exhibited a significant decrease in the first 6 epochs and began to stabilize around the 75th epoch, indicating that the model was gradually converging. After 75 epochs, the loss value remained largely unchanged, indicating that the model was approaching convergence. The relatively quick convergence of the model can be attributed to the small sample size and the simple mapping relationship between the UV-Vis spectra and the COD values, which facilitated the learning process. Despite the model having converged, the final loss value only decreased to around 15, rather than reaching 0, which can be attributed to factors such as noise and turbidity in the spectra, which introduced some interference during training.

### 3.3. Visualization of Feature Importance

**Figure 7:**
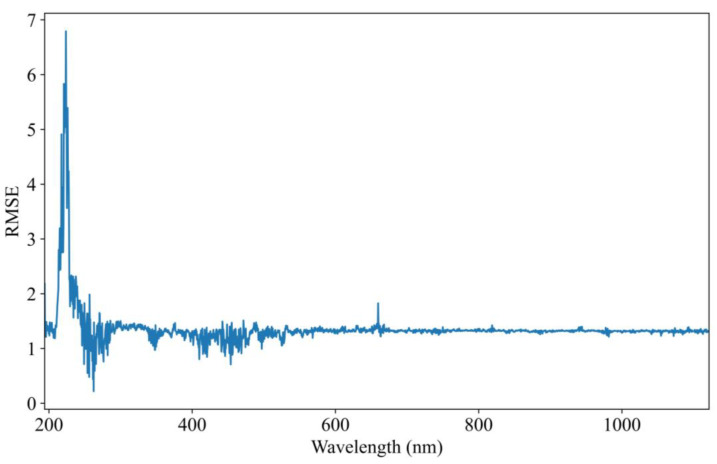
UV-Vis spectral importance curve based on permutation feature importance.

The evaluation of feature importance is essential for understanding the contribution of each input variable to COD prediction performance. This study assesses the significance of individual features within the trained model’s prediction set. Identifying key features enhances model interpretability and helps optimize input selection for improved accuracy and efficiency. To quantify feature importance, the permutation feature importance (PFI) method was employed. PFI is a model-agnostic technique that measures the influence of each feature by randomly shuffling its values and observing the corresponding change in model performance. A greater increase in prediction error indicates a more influential feature, while minimal change suggests a less significant contribution. RMSE is used as the primary evaluation metric. The RMSE quantifies the deviation between predicted and actual COD values, providing an objective measure of model performance. Higher RMSE values after feature permutation indicate a stronger dependence of the model on the corresponding feature. To facilitate analysis, the feature importance results are visualized in Figure 7. This graphical representation highlights the relative significance of each feature, offering valuable insights into their role in COD prediction.

The feature importance analysis reveals a strong correlation between the spectral feature importance curve (Figure 7) and the absorption spectrum (Figure 2). Notably, the most critical spectral region for COD prediction aligns with the main absorption peak around 250 nm, which is clearly visible in Figure 2. This indicates that the model is effectively identifying and prioritizing the relevant features that are crucial for accurate COD prediction. The model’s ability to automatically extract features related to COD further confirms its capacity to learn and adapt to important spectral patterns. By focusing on the main absorption peak, the model captures essential information that directly correlates with COD levels in the water samples. However, it is also observed that the model occasionally learns irrelevant features, such as the 660 nm peak, which is likely a result of spectrometer noise rather than any meaningful COD-related information. This extraction of irrelevant features can negatively influence the model’s accuracy. The inclusion of such noise introduces unwanted variability into the prediction process, reducing the model’s precision in detecting COD concentrations. Moreover, non-COD-related factors, such as turbidity and organic salts, may introduce further interference in the UV-Vis spectroscopy data. These external factors contribute to inaccuracies in the COD detection process, highlighting the need for the careful consideration of potential confounding variables when training and deploying the model for real-world applications.

### 3.4. Model Performance Analysis

**Figure 8:**
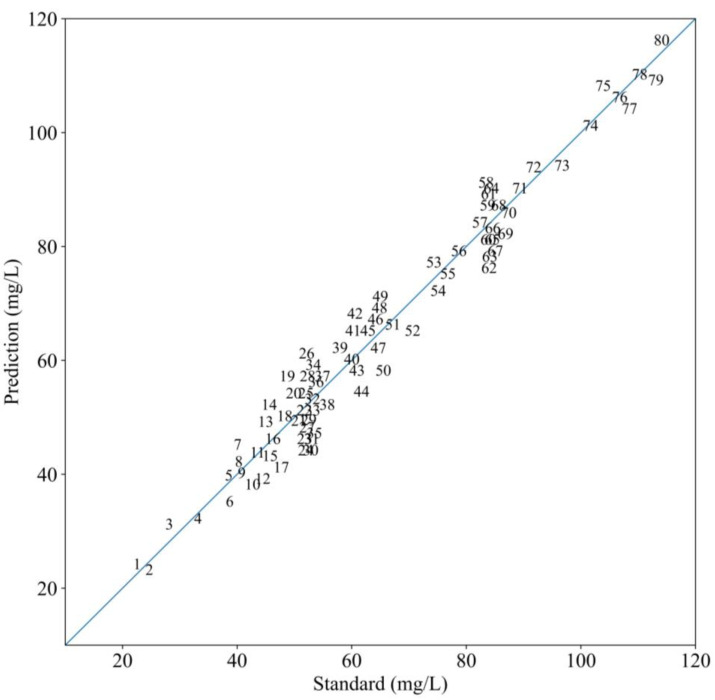
Comparison curve between the prediction and standard COD values.

After the training stage, the model that achieved the lowest loss value was saved as the final COD prediction model, ensuring that the most optimized version of the model was employed for further testing. The saved model was then tested by the 80 testing/prediction set. The predicted COD values from the model were compared with the standard COD values to assess the model’s performance. A summary of the model’s performance, including various evaluation metrics, is provided in Table 9. To quantify the accuracy of the model’s predictions, a linear analysis was conducted by comparing the predicted COD values with the standard COD values. The results of this linear fitting analysis are shown in Figure 8.

The linearity between the predicted and standard values is shown in Figure 8, with higher accuracy indicated by the scatter points being closer to the fitted line. The strong agreement between the predicted and standard values suggests that the model possesses good robustness and adaptability, making it highly reliable for accurate COD prediction in water samples. Specifically, the MS-1D-CNN model demonstrated a clear linear relationship between the predicted and standard COD values, with the scatter points closely following the straight line that has a slope of 1. Importantly, no significant error growth was observed as the COD concentration increased, indicating that the model performs consistently across a wide range of COD concentrations, particularly between 20 and 120 mg/L. This stability in error distribution further highlights the generalization ability of the model. The experiments confirm that the method effectively employs spectral information from across the entire UV–visible spectrum, ensuring comprehensive feature extraction for precise COD predictions.

### 3.5. Comparison with Other Methods

**Figure 9:**
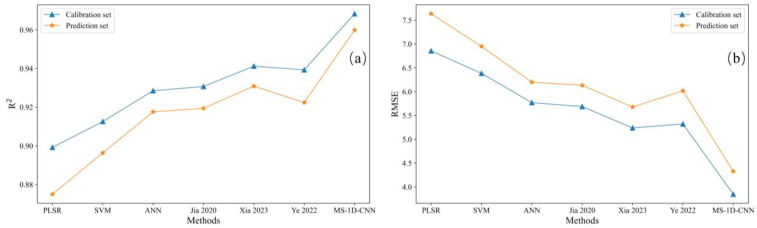
Comparison of the proposed method (**a**) and traditional methods (**b**). Here, Jia 2020 represents 1D-CNN [19], Xia 2023 represents 1D-CNN [22], Ye 2022 represents 1D-CNN [26].

To further evaluate the effectiveness of the proposed method, some traditional methods were used as a comparison. The performance of the proposed model was thoroughly evaluated by comparing its prediction and fitting accuracy with that of three widely recognized traditional methods and deep learning methods for COD prediction. Traditional methods included PLS [34], SVM [35], and artificial neural network (ANN) [36]. Deep learning methods included three 1D-CNNss [19,22,26]. These methods were selected due to their frequent application in similar predictive modeling tasks. The comparison of the models’ performance was based on the same performance indices, which are crucial for assessing their ability to accurately predict COD values. The detailed results of the comparison are presented in Table 10, which summarizes the performance of each model. Additionally, the graphical representation of these results is shown in Figure 9, providing a visual comparison that facilitates a better understanding of the relative strengths and weaknesses of each model. This comparison aims to demonstrate the effectiveness of the proposed method and its potential advantages over traditional methods in COD prediction tasks.

The comparison of methods, as shown in Table 10 and Figure 9, reveals clear distinctions in performance between the proposed method, traditional methods, and deep learning models. Among the traditional methods, ANN shows the best performance with an R^2^ of 0.9286/0.9176. However, it still falls short of fully fitting the nonlinear relationship between spectra and COD. Its fitting performance is inferior to that of the proposed method. The other two traditional methods, PLS and SVM, exhibit even poorer fitting results. Meanwhile, ANN has lower RMSEC/RMSEP values (5.7691/6.1994), suggesting smaller prediction errors. Both PLS and SVM also exhibit inferior performance, with higher RMSEC/RMSEP, indicating that these methods have lower accuracy in COD prediction. Turning to deep learning methods, the 1D-CNN model referenced in previous studies [22] delivers the best results among deep learning approaches, with an R^2^ of 0.9412/0.9309 and RMSEC/RMSEP values of 5.2387/5.6779. However, the other two deep learning models in comparison perform worse than the 1D-CNN, demonstrating their relative inefficiency in capturing the spectral–COD relationship. The proposed method outperforms all these traditional and deep learning models, including PLS, SVM, ANN, and the three 1D-CNN models. It achieves a superior goodness of fit, with R^2^ values of 0.9683/0.9599, which is significantly higher than those of the other methods. This indicates that the proposed method has a stronger capability to capture the complex nonlinear relationship between the spectral data and COD values. Moreover, the proposed method demonstrates a remarkable level of accuracy, with much lower RMSEC and RMSEP values of 3.8464/4.3259, confirming its exceptional precision in COD prediction. In addition to its accuracy, the robustness of the proposed method is evident. It not only provides better fitting results but also maintains high prediction accuracy, highlighting its ability to effectively extract meaningful features from the spectral data and deliver reliable COD predictions.

## 4. Discussion

The proposed model demonstrates its effectiveness in COD detection using UV-Vis spectroscopy, as evidenced by the performance comparison presented in Table 10. The model’s prediction accuracy is reflected in the RMSEC and RMSEP values of 3.8464 and 4.3259, respectively, indicating strong precision in predicting COD values. Additionally, the R^2^ values of 0.9683 for the calibration set and 0.9599 for the prediction set highlight the model’s excellent fitting performance, confirming its ability to capture the complex relationships between spectral data and COD. Further, the comparison with other models in the literature emphasizes the superiority of the proposed multi-parallel CNNs. The three-parallel model, which utilizes three separate 1D-CNNs, outperforms the two-parallel model from [22] as well as the single-model architectures presented in [19,26]. This demonstrates the added advantage of using multiple parallel networks for feature extraction and model performance. The results also underline the significant advantages of deep learning methods over traditional techniques, particularly in the domain of COD detection. Deep learning models, like the proposed one, excel in capturing complex, nonlinear relationships within the data, making them far more effective than traditional methods such as ANN, SVM, and PLS. This reinforces the efficacy of deep learning-based approaches for accurate and reliable COD prediction.

The model presented in this paper significantly improves the accuracy of COD detection using UV-Vis spectroscopy. However, the model structure is not entirely optimal, as the parameters were selected based on prior experience and may require further optimization for better performance. The model shows limitations in terms of generalization, particularly when applied to other surface waters or industrial wastewater, where the prediction accuracy may not be as high. This is because the model has been trained using a specific dataset, and it struggles to infer the mapping relationship for data outside the scope of the current study. To address these limitations, it is essential to fine-tune the model for use in other water bodies. This would involve collecting corresponding samples from different water sources and adjusting the model accordingly. Such fine-tuning would help improve the model’s generalization capabilities and ensure accurate COD prediction in diverse environments. Therefore, while the current model demonstrates high effectiveness within the scope of the data used, its broader application will require additional adjustments to enhance its robustness across varying water types.

## 5. Conclusions

The primary objective of this paper is to address the issue of inaccurate COD prediction based on UV-Vis spectroscopy. To overcome this limitation, a novel COD prediction model is proposed, based on a multi-scale one-dimensional convolutional neural network (MS-1D-CNN). This model is designed to enhance the accuracy of COD detection by effectively extracting relevant features from spectral data. The feature extraction process is performed by three parallel sub-one-dimensional convolutional neural networks, each operating at different scales. These extracted features are then fused through element-wise addition, combining the information from multiple scales into a comprehensive feature representation. The final COD results are output through fully connected layers after the feature fusion process. The fusion neural network model offers several advantages, as it allows for a more complete characterization of the sample’s absorption spectrum, improving the model’s ability to capture critical information. The multi-scale approach significantly enhances the accuracy of COD predictions by leveraging information from various spectral scales. The effectiveness of this method was validated through carefully designed experiments, which demonstrated that the MS-1D-CNN model is highly successful in extracting and quantifying characteristic information from absorption spectra. Moreover, the MS-1D-CNN model outperforms traditional COD prediction methods and other deep learning methods, in terms of regression accuracy. This method reduces the reliance on conventional spectral preprocessing techniques by employing the powerful feature extraction capabilities of the multi-scale 1D-CNN. Overall, the research indicates that the proposed method leads to improved fitting and higher prediction accuracy, providing an effective solution for rapid and accurate COD detection.

The method presented in this study has significant potential for real-time water quality measurement. By leveraging UV-Vis spectroscopy and the proposed deep learning model, this approach allows for the continuous monitoring of COD in water, providing up-to-date data on water quality. Such real-time monitoring can be invaluable for governments and environmental agencies, as it supports the formulation of effective environmental policies. With accurate and timely information on pollution levels, authorities can take proactive measures to safeguard water resources and protect the ecological environment. However, for successful deployment, the model may require fine-tuning based on local water samples. In practical applications, variations in water composition may lead to a decrease in detection accuracy. To address this, the model should be adjusted and retrained using localized data to ensure optimal performance and maintain high accuracy across different water sources. This fine-tuning process is crucial for enhancing the model’s robustness and ensuring its effectiveness in real-world environmental monitoring applications.

## Figures and Tables

**Table 1:** COD statistical results of the sample set.

Sample Set	Samples	Mean (mg/L)	Minimum (mg/L)	Maximum (mg/L)	Standard Deviation (mg/L)
Training set	400	64.58	21.1	115.2	21.57
Testing set	80	64.71	22.5	114.1	21.60
All	480	64.60	21.1	115.2	21.58

**Table 2:** The influence of convolutional kernel size on the performance of 1D-CNN.

Convolutional Kernel Size	Calibration Set		Prediction Set	
	R^2^	RMSEC	R^2^	RMSEP
1	0.8495	8.3780	0.8371	8.7163
3	0.8506	8.3484	0.8394	8.6546
5	0.8539	8.2556	0.8491	8.3884
7	0.8477	8.4283	0.8359	8.7492
9	0.8434	8.5463	0.8289	8.9327
11	0.8396	8.6497	0.8274	8.9732
21	0.8334	8.8149	0.8188	9.1931
51	0.8346	8.7837	0.8206	9.1468
101	0.8405	8.6247	0.8319	8.8555

**Table 3:** The influence of the number of convolutional kernels on the performance of 1D-CNN.

Number of Convolutional Kernels	Calibration Set		Prediction Set	
	R^2^	RMSEC	R^2^	RMSEP
8	0.8514	8.3256	0.8444	8.5183
16	0.8558	8.2016	0.8468	8.4545
32	0.8569	8.1710	0.8491	8.3898
64	0.8535	8.2670	0.8485	8.4069
128	0.8497	8.3722	0.8354	8.7633
256	0.8469	8.4509	0.8297	8.9117
512	0.8477	8.4285	0.8326	8.8371

**Table 4:** The influence of the number of convolutional layers on the performance of 1D-CNN.

Number of Convolutional Layers	Calibration Set		Prediction Set	
	R^2^	RMSEC	R^2^	RMSEP
1	0.8360	8.7474	0.8165	9.2521
2	0.8524	8.2978	0.8375	8.7054
3	0.8532	8.2760	0.8397	8.6478
4	0.8514	8.3241	0.8331	8.8237
5	0.8461	8.4726	0.8284	8.9460
6	0.8458	8.4816	0.8260	9.0086
7	0.8506	8.3470	0.8353	8.7640
8	0.8491	8.3893	0.8235	9.0721
9	0.8523	8.2993	0.8308	8.8843
10	0.8513	8.3279	0.8318	8.8562

**Table 5:** The influence of the number of neurons in the fully connected layers on the performance of 1D-CNN.

Number of Neurons in the Fully Connected Layers	Calibration Set		Prediction Set	
	R^2^	RMSEC	R^2^	RMSEP
16	0.8428	8.5635	0.8268	8.9871
32	0.8579	8.1418	0.8398	8.6442
64	0.8536	8.2642	0.8360	8.7451
128	0.8209	9.1400	0.8114	9.3792
256	0.8589	8.1119	0.8417	8.5938
512	0.8483	8.4109	0.8284	8.9452
1024	0.8178	9.2176	0.8052	9.5320
4096	0.8239	9.0632	0.8121	9.3614

**Table 6:** The influence of the number of fully connected layers on the performance of 1D-CNN.

Number of Fully Connected Layers	Calibration Set		Prediction Set	
	R^2^	RMSEC	R^2^	RMSEP
1	0.8545	8.2386	0.8376	8.7031
2	0.8717	7.7351	0.8622	8.0174
3	0.8463	8.4674	0.8253	9.0272
4	0.8017	9.6181	0.7907	9.8795
5	0.8221	9.1098	0.8188	9.1931

**Table 7:** Parameters of the proposed MS-1D-CNN architecture.

Layer	Input Shape	Output Shape	Kernel Number	Kernel Size	Padding	Stride	Activation
Convolution 0_1	(1, 2048, 1)	(1, 2048, 16)	16	1 × 1	0	1	ReLU
Convolution 1_1	(1, 2048, 1)	(1, 2048, 16)	16	1 × 3	1	1	ReLU
Convolution 2_1	(1, 2048, 1)	(1, 2048, 16)	16	1 × 5	2	1	ReLU
Max Pooling 0_1	(1, 2048, 16)	(1, 1024, 16)	None	1 × 2	None	2	None
Max Pooling 1_1	(1, 2048, 16)	(1, 1024, 16)	None	1 × 2	None	2	None
Max Pooling 2_1	(1, 2048, 16)	(1, 1024, 16)	None	1 × 2	None	2	None
Convolution 0_2	(1, 1024, 16)	(1, 1024, 32)	32	1 × 1	0	1	ReLU
Convolution 1_2	(1, 1024, 16)	(1, 1024, 32)	32	1 × 3	1	1	ReLU
Convolution 2_2	(1, 1024, 16)	(1, 1024, 32)	32	1 × 5	2	1	ReLU
Max Pooling 0_2	(1, 1024, 32)	(1, 512, 32)	None	1 × 2	None	2	None
Max Pooling 1_2	(1, 1024, 32)	(1, 512, 32)	None	1 × 2	None	2	None
Max Pooling 2_2	(1, 1024, 32)	(1, 512, 32)	None	1 × 2	None	2	None
Convolution 0_3	(1, 512, 32)	(1, 512, 64)	64	1 × 1	0	1	ReLU
Convolution 1_3	(1, 512, 32)	(1, 512, 64)	64	1 × 3	1	1	ReLU
Convolution 2_3	(1, 512, 32)	(1, 512, 64)	64	1 × 5	2	1	ReLU
Max Pooling 0_3	(1, 512, 64)	(1, 256, 64)	None	1 × 2	None	2	None
Max Pooling 1_3	(1, 512, 64)	(1, 256, 64)	None	1 × 2	None	2	None
Max Pooling 2_3	(1, 512, 64)	(1, 256, 64)	None	1 × 2	None	2	None
Fusion	(1, 256, 64)	(1, 256, 64)	None	None	None	None	None
Flatten	(1, 256, 64)	(1, 16384)	None	None	None	None	None
FC	(1, 16384)	(1, 256)	None	None	None	None	ReLU
FC	(1, 256)	(1, 32)	None	None	None	None	ReLU
FC (Output)	(1, 32)	(1, 1)	None	None	None	None	None

**Table 8:** The performance on the training set with the combination of learning rate and batch size.

Learning Rate	Batch Size									
	8		16		32		64		128	
	R^2^	RMSEC	R^2^	RMSEC	R^2^	RMSEC	R^2^	RMSEC	R^2^	RMSEC
0.00005	0.9226	6.0091	0.9345	5.5288	0.9430	5.1573	0.9501	4.8256	0.9395	5.3105
0.0001	0.9181	6.1791	0.9248	5.9214	0.9681	3.8567	0.9386	5.3515	0.9291	5.7506
0.0005	0.9288	5.7608	0.9497	4.8428	0.9388	5.3426	0.9468	4.9811	0.9440	5.1092
0.001	0.9399	5.2932	0.9115	6.4251	0.9338	5.5578	0.9492	4.8671	0.9492	4.8686
0.003	0.9491	4.8733	0.9326	5.6051	0.9420	5.2006	0.9333	5.5770	0.9328	5.5988
0.005	0.9286	5.7691	0.9379	5.3801	0.9324	5.6159	0.9428	5.1656	0.9211	6.0676
0.01	0.9200	6.1097	0.9269	5.8407	0.9470	4.9727	0.9297	5.7250	0.9189	6.1494

**Table 9:** The performance of the MS-1D-CNN model.

Method	Calibration Set		Prediction Set	
	R^2^	RMSEC	R^2^	RMSEP
MS-1D-CNN	0.9683	3.8464	0.9599	4.3259

**Table 10:** Comparison of the proposed method and traditional methods.

Method	Calibration Set		Prediction Set	
	R^2^	RMSEC	R^2^	RMSEP
PLSR	0.8992	6.8557	0.8750	7.6350
SVM	0.9126	6.3840	0.8964	6.9516
ANN	0.9286	5.7691	0.9176	6.1994
1D-CNN [19]	0.9307	5.6863	0.9194	6.1326
1D-CNN [22]	0.9412	5.2387	0.9309	5.6779
1D-CNN [26]	0.9393	5.3215	0.9224	6.0175
MS-1D-CNN	0.9683	3.8464	0.9599	4.3259

## Data Availability

The data that support the findings of this study are available from the corresponding author.
